# Integrative Clinical, Molecular, and Computational Analysis Identify Novel Biomarkers and Differential Profiles of Anti-TNF Response in Rheumatoid Arthritis

**DOI:** 10.3389/fimmu.2021.631662

**Published:** 2021-03-23

**Authors:** Maria Luque-Tévar, Carlos Perez-Sanchez, Alejandra Mª Patiño-Trives, Nuria Barbarroja, Ivan Arias de la Rosa, Mª Carmen Abalos-Aguilera, Juan Antonio Marin-Sanz, Desiree Ruiz-Vilchez, Rafaela Ortega-Castro, Pilar Font, Clementina Lopez-Medina, Montserrat Romero-Gomez, Carlos Rodriguez-Escalera, Jose Perez-Venegas, Mª Dolores Ruiz-Montesinos, Carmen Dominguez, Carmen Romero-Barco, Antonio Fernandez-Nebro, Natalia Mena-Vazquez, Jose Luis Marenco, Julia Uceda-Montañez, Mª Dolores Toledo-Coello, M. Angeles Aguirre, Alejandro Escudero-Contreras, Eduardo Collantes-Estevez, Chary Lopez-Pedrera

**Affiliations:** ^1^Instituto Maimónides de Investigación Biomédica de Córdoba (IMIBIC), Hospital Reina Sofia, Universidad de Cordoba, Córdoba, Spain; ^2^Hospital Universitario de Jaen, Jaén, Spain; ^3^Hospital Universitario Virgen Macarena, Sevilla, Spain; ^4^Hospital Clínico Universitario, Malaga, Spain; ^5^Hospital Regional Universitario de Malaga, Malaga, Spain; ^6^Hospital Universitario Virgen de Valme, Sevilla, Spain; ^7^Hospital Universitario de Jerez de la Frontera, Cádiz, Spain

**Keywords:** rheumatoid arthritis, anti-TNF agents, inflammation, NEtosis, microRNAs, machine learning, predictors, efficacy

## Abstract

**Background:** This prospective multicenter study developed an integrative clinical and molecular longitudinal study in Rheumatoid Arthritis (RA) patients to explore changes in serologic parameters following anti-TNF therapy (TNF inhibitors, TNFi) and built on machine-learning algorithms aimed at the prediction of TNFi response, based on clinical and molecular profiles of RA patients.

**Methods:** A total of 104 RA patients from two independent cohorts undergoing TNFi and 29 healthy donors (HD) were enrolled for the discovery and validation of prediction biomarkers. Serum samples were obtained at baseline and 6 months after treatment, and therapeutic efficacy was evaluated. Serum inflammatory profile, oxidative stress markers and NETosis-derived bioproducts were quantified and miRNomes were recognized by next-generation sequencing. Then, clinical and molecular changes induced by TNFi were delineated. Clinical and molecular signatures predictors of clinical response were assessed with supervised machine learning methods, using regularized logistic regressions.

**Results:** Altered inflammatory, oxidative and NETosis-derived biomolecules were found in RA patients vs. HD, closely interconnected and associated with specific miRNA profiles. This altered molecular profile allowed the unsupervised division of three clusters of RA patients, showing distinctive clinical phenotypes, further linked to the TNFi effectiveness. Moreover, TNFi treatment reversed the molecular alterations in parallel to the clinical outcome. Machine-learning algorithms in the discovery cohort identified both, clinical and molecular signatures as potential predictors of response to TNFi treatment with high accuracy, which was further increased when both features were integrated in a mixed model (AUC: 0.91). These results were confirmed in the validation cohort.

**Conclusions:** Our overall data suggest that: 1. RA patients undergoing anti-TNF-therapy conform distinctive clusters based on altered molecular profiles, which are directly linked to their clinical status at baseline. 2. Clinical effectiveness of anti-TNF therapy was divergent among these molecular clusters and associated with a specific modulation of the inflammatory response, the reestablishment of the altered oxidative status, the reduction of NETosis, and the reversion of related altered miRNAs. 3. The integrative analysis of the clinical and molecular profiles using machine learning allows the identification of novel signatures as potential predictors of therapeutic response to TNFi therapy.

## Introduction

Rheumatoid arthritis (RA) is a systemic inflammatory autoimmune disease identified by continuous joint inflammation promoting cartilage and bone damage, incapacity and eventually systemic complications. Prompt treatment can preclude severe disability and bring significant benefits to patients, although the lack of therapeutic efficacy in a substantial number of patients is still challenging (1).

In the last years, advances in the understanding of RA pathogenesis by identifying key cells and cytokines have allowed the development of new targeted disease-modifying antirheumatic drugs (2). In the late 1990s, the introduction of anti-tumor necrosis factor alpha drugs (TNF inhibitors, TNFi) greatly improved the medical management of RA patients, although some of them were reported to be ineffective.

A recent observational study has found that nowadays anti-TNF drugs are the first-line treatment in 96% of patients who fail methotrexate therapy. Besides, patients who do not reach their treatment targets (3) are forced to cycle through multiple anti-TNF drugs while their disease has time to progress. As all anti-TNF drugs target similar molecular and inflammatory pathways, it is not surprising that most patients who are primary non-responders to their initial anti-TNF therapy fail to achieve their treatment targets when cycled through alternative anti-TNFs. This suggests that primary non-responders should be switched to an alternative therapy rather than enduring anti-TNF cycling. Thus, the development of a personalized medicine approach to identify primary non-responders to anti-TNFs prior to treatment would allow significantly more patients to reach their treatment target by treating them with alternative therapies as first-line therapies.

Nowadays, the number of robust treatment response predictors in RA is very limited, so that it has been demonstrated that pathophysiological biomarkers have insufficient discriminating power. Hence, several studies have identified a number of potential clinical biomarkers of RA response to biological therapies, including age, sex, disease duration and activity, smoking status, presence of comorbidities, tender joint counts (TJC), concomitant methotrexate therapy, etc. (4–7). Yet, those studies were inconsistent and contradictory results have been published.

Besides, none of these studies have evaluated the molecular mechanisms underlying the distinctive response to TNF-inhibitors (TNFi) among RA patients and their potential as predictors of treatment response.

In the last years, relevant findings in the field of RA pathogenesis have been described, among which new insights come from studies on synovial fibroblasts and cells belonging to the innate and adaptive immune system, which have documented the aberrant production of inflammatory mediators, oxidative stress and NETosis, along with relevant alterations of the genome and on the regulatory epigenetic and posttranslational mechanisms. Moreover, emerging studies by several groups, including ours, have demonstrated that the pharmacological therapy with biological disease modifying anti-rheumatic drugs (bDMARDs) such as TNF or IL-6 receptor inhibitors, or anti-CD20 antibodies promotes, in parallel to their clinical efficacy, a specific and significant alteration in several of these altered molecular mechanisms (8–12).

The complexity of the treatment response in a given patient and the significant differences between patients suggest that the combination of biomarkers may be more helpful than studying them separately. Therefore, the development of matrices containing clinical and laboratory parameters related to diagnosis or prognosis might help to select the best treatment for each patient.

Integrative biology by advanced computational analysis is a fast-expanding field that can be expected to identify combinations of parameters capable of predicting the response to various drugs (13).

In this study, we developed an integrative clinical and molecular longitudinal study in RA patients to explore changes in serologic parameters related to inflammation, NETosis, oxidative stress and regulating microRNAs (miRNAs) following TNFi treatment. Besides, by using machine-learning algorithms, we aimed at the prediction of TNFi response based on the combination of clinical and molecular profiles of RA patients.

## Patients and Methods

### Study Design and Patients

In a prospective multicenter study, a total of 104 RA patients and 29 healthy donors (HD), from two independent cohorts, were recruited (during a 48-months period). These cohorts attended the Reina Sofia University Hospital of Córdoba, the Virgen Macarena Hospital of Sevilla, The Virgen del Valme Hospital of Sevilla, the Virgen de la Victoria University hospital of Malaga, Jerez de la Frontera University Hospital, and the University Hospital of Jaen. All patients fulfilled the American College of Rheumatology revised criteria for RA (14).

Approval from the ethics committees was obtained, and subjects provided written informed consent.

Clinical/laboratory parameters of RA patients and HD from the discovery cohort are displayed in Table 1 while clinical data of RA patients belonging to the independent validation cohort are displayed in Supplementary Table 1.

**Table 1:** Clinical and molecular profiles of rheumatoid arthritis patients and healthy donors recruited to the study.

	**HD (*n* = 29)**	**RA patients (*n* = 79)**	***p***
**Clinical parameters**			
Gender (female/male)	19/10	64/15	0.12
Age, years (mean ± SD)	47 ± 17	51.2 ± 10.5	0.056
Disease evolution, years (mean ± SD)		11.5 ± 9.1	
TJC (mean ± SD)		8.1 ± 6.0	
SJC (mean ± SD)		5.9 ± 4.9	
DAS28 (mean ± SD)		4.7 ± 1.2	
SDAI (mean ± SD)		29.6 ± 13.3	
CDAI (mean ± SD)		27.9 ± 11.9	
HAQ (mean ± SD)		1.4 ± 0.7	
Smoking (*n*, %)	5/29 (17%)	19/79 (24%)	0.323
Arterial hypertension (*n*, %)	0/29 (%)	17/79 (21%)	0.005
Diabetes (*n*, %)	0/29 (%)	7/79 (8%)	0.186
Hypercholesterolemia (*n*, %)	16/29 (%)	35/79 (44%)	0.197
Extra-articular manifestations (*n*, %)		13/79 (16%)	
Radiological involvement (*n*, %)		29/79 (37%)	
Eroded joints (mean ± SD)		1.3 ± 2.3	
**Laboratory parameters**			
CRP, mg/mL (mean ± SD)	1.6 ± 2.2	15.8 ± 26.2	0.000
ESR, mm/h (mean ± SD)	8.1 ± 5.8	23.7 ± 17.9	0.000
ACPAs, IU/mL (mean ± SD)		343.3 ± 762.6	
RF, IU/mL (mean ± SD)		112.9 ± 205.8	
**Treatments**			
NSAIDS (*n*, %)		59/79 (74%)	
Corticosteroids (*n*, %)		73/79 (92%)	
Statins (*n*, %)		8/79 (10%)	
Vit D (*n*, %)		24/79 (30%)	
Osteoporosis treatment (*n*, %)		18/79 (22%)	
Antiplatelet (*n*, %)		2/79 (2%)	
Anticoagulants (*n*, %)		2/79 (2%)	
Methotrexate (*n*, %)		48/79 (61%)	
Leflunomide (*n*, %)		50/79 (63%)	
Dolquine (*n*, %)		46/79 (58%)	
Salazopyrine (*n*, %)		16/79 (20%)	

All patients had an inadequate response to at least two disease-modifying antirheumatic drugs (DMARDs) and received TNFi in combination therapy with DMARDs. All patients were naïve to TNFi treatment.

Within the discovery cohort, 22 patients were given infliximab (3 mg/kg/day intravenous infusion at times 0, 2, and 6 weeks, and every 8 weeks thereafter), 39 patients received etanercept (50 mg subcutaneously every week), 12 patients were treated with adalimumab (40 mg subcutaneously every 2 weeks), 4 with golimumab (50 mg subcutaneously every month) and 2 with certolizumab (400 mg at times 0, 2, and 4 weeks, and 200 mg every 2 weeks thereafter).

Within the validation cohort, 5 patients were given infliximab, 10 patients received etanercept, 8 patients were treated with adalimumab, 1 with golimumab and 1 with certolizumab.

Clinical assessment, before and after 3 and 6 months of TNFi therapy, included swollen joint count (SJC), tender joint count (TJC), 28-joint disease activity score (DAS28), clinical disease activity index (CDAI), simple disease activity index (SDAI), and health assessment questionnaire (HAQ). Serological evaluation, performed by clinical laboratory routine analysis, included analysis of rheumatoid factor (RF, U/mL), anti-cyclic citrullinated peptide antibodies (ACPAs, U/mL), C-reactive protein (CRP, mg/L) and erythrocyte sedimentation rate (ESR, mm/h).

Response to TNFi treatment was assessed by the European League Against Rheumatism (EULAR) criteria, based on the changes in DAS28 score, and the patients were categorized into responders and non-responders to TNFi. An improvement in DAS28 over ≥1.2 and a DAS28 value ≤ 3.2 after 6 months of treatment was considered a good response; a DAS28 value after 6 months between 3.2 and 5.1 and a reduction between 0.6 and 1.2 was considered a moderate response. Both of them were categorized as responders to the therapy. Patients who exhibited a DAS28 score at T6 > 5.1 or a reduction in DAS28 under 0.6 were considered a non-responders.

### Blood Collection

Whole blood from HD and RA patients was collected by direct venous puncture either, into tubes with ethylenediaminetetraacetic acid (EDTA) as an anticoagulant, or into specific tubes for obtaining serum.

Blood samples were obtained before and after 6 months of TNFi treatment. To avoid blood composition changes promoted by diet and circadian rhythms, samples were always collected in the early hours in the morning and after a fasting period of 8 h. The blood was processed by spinning at 2,000 × g for 10 min at room temperature. Then, serum was transferred to a fresh RNase-free tube and stored at −80°C.

To avoid differences related to the origin of samples and their processing all the blood samples were collected and processed following the same protocol. In this multicenter study, our lab was the reference center. Thus, tubes for obtaining blood were sent to all the hospitals that collaborated on recruitment, and serum purification and storage were developed under the same conditions. Hence, all the samples coming from external centers were processes in our lab following the same procedures, which ensured the homogeneity of the downstream analysis.

### Assessment of Circulating Inflammatory Profile and Oxidative Stress Markers

The inflammatory profile was analyzed in the serum of HD and RA patients both, before and after 6 months of TNFi therapy, by using a multiplex-type immunoassay—Bioplex (*Bio-Rad, CA, USA*)—in which a panel of 27 cytokines was evaluated.

Oxidative stress parameters were determined through the evaluation of oxidation of both lipids and proteins, along with the analysis of the total antioxidant capacity. Assays of lipid peroxidation levels were carried out using the Thiobarbituric acid reactive substances (TBARS) assay kit (Canvax Biotech, Córdoba, Spain), following the manufacturer's recommendations.

Protein nitrosylation was measured by using the Nitrotyrosine ELISA kit (Abcam, Cambridge, UK), following the manufacturer's recommendations. Serum total antioxidant capacity (TAC) was measured by quantitative colorimetric determination, using TAC Assay kit (Biovision, Mountain View, CA, USA) following the instructions provided by the manufacturer.

### NETosis-Derived Products Assessment

To analyze NETosis-derived products, circulating levels of both elastase and nucleosomes were evaluated. Cell-free elastase levels were measured in RA patients' and HDs' serum using the Human PMN Elastase ELISA Kit (Abcam, Cambridge, UK) following the manufacturer's recommendations.

Likewise, cell-free nucleosomes were measured using the human cell death detection ELISAPLUS kit (Roche, Sigma-Aldrich, St Louis, MO, USA) following the manufacturer's recommendations. In this assay, monoclonal antibodies against DNA (double and single strand) and histones (H1, H2A, H2B, H3, and H4) were used to detect mono- and oligo nucleosomes in serum from RA patients. Quantification of nucleosomes was performed by photometrical determination of the absorbance at 405 nm, using as reference wavelength 492 nm.

### MicroRNA Isolation, Profiling, and Quantitative Real-Time PCR

Total serum RNA—including the miRNA fraction—was extracted using the QIAzol miRNeasy kit (*Qiagen, Valencia, CA, USA*) with some modifications. A total of 200 μl of serum were thawed on ice and lysed in 1 mL QIAzol Lysis Reagent (*Qiagen*). Samples in QIAzol were incubated at room temperature for 5 min to inactivate RNases. To adjust for variations in RNA extraction and/or copurification of inhibitors, 5 fmol of spike-in non-human synthetic miRNA (*C. elegans* miR-39 miRNA mimic: 5′-UCACCGGGUGUAAAUCAGCUUG-3′) were added to the samples after the initial denaturation. The remaining extraction protocol was performed according to the manufacturer's instruction. Total RNA was eluted in 14 μl of RNase-free water.

To identify the profiles of miRNAs in the serum of HDs and RA patients, an array was performed in an exploratory cohort -including 6 samples from clinically representative RA patients and 3 from HDs- using the HTG EdgeSeq miRNA whole transcriptome assay (*miRNA WTA*), which enabled to measure the expression of 2,083 human miRNA transcripts using next generation sequencing (NGS) (HTG Molecular technologies, Tucson, AZ, USA).

All differentially regulated miRNAs and fold changes were imported into the web-based bioinformatics tool QIAGEN's Ingenuity Pathway Analysis (IPA) (Ingenuity Systems, http://www.INGENUITY.com) to perform a functional classification and identify potential mRNA targets. The right-tailed Fisher's exact test was used to calculate the *p*-value determining the statistical probability that the association between a set of molecules and a pathway or function might be due to chance alone. IPA analysis also allowed the selection of altered miRNAs that exhibited an enrichment in mRNA targets involved in the pathogenesis of RA for their validation in the whole cohort by real time PCR (RT-PCR) using a LightCycler® Thermal Cycler System (Roche Diagnostics, Indianapolis, Indiana, USA). Specifically, 3 μl of RNA eluate were reverse transcribed in 10 μl reactions using the miRCURY LNATM Universal RT mi-RNA PCR, Polyadenylation and cDNA synthesis kit (Exiqon, Vedbaek, Denmark). RT-PCR was carried out with 4 μL cDNA diluted 20x and 6 μL of reaction mixture [5 μL of SYBR Green master mix (Exiqon) and 1 μL of the corresponding PCR primer mix (microRNAs LNATM PCR primer set, Exiqon)]. After an initial hold of 10 min at 94°C, samples were cycled 40 times at 95°C for 10 s and at 60°C for 1 min. The expression levels of miRNAs were normalized to the mean of spiked-in miRNA Cel-miR-39. The expression levels of miRNA were calculated using the 2-ΔΔCt method. All measurements were performed in duplicate. Controls consisting of reaction mixture without cDNA were negative in all runs. List of miRNA sequences is displayed in Supplementary Table 2.

### Machine Learning Analysis

Three different logistic models (15) were made to study clinical and molecular variables groups before starting therapy, looking for patients' best classification as responders or non-responders, using Python library Scikit-Learn (16). A logistic regression model with L2 penalty (all variables used) was made for clinical variables group. Training set (75%) and test set (25%) were settled for model validation. Identical approach was developed for molecular variables group. To study combined effect of both variables' groups, the same approach was used changing only to L1 penalty (variables selection) (Supplementary Figure 1).

### Statistical Analyses

Statistical analysis and graphical representation of results were performed using GraphPad Prism 8 software (San Diego CA, USA). The normal distribution of the variables to characterize the differences in the analyzed parameters was assessed using the Kolmogorov-Smirnov test.

Based on this test, comparisons between quantitative and qualitative variables were made using the Student's *t*-test, or alternatively, using a non-parametric test (Mann-Whitney U).

Paired samples within the same subjects were compared by Wilcoxon signed-rank test. Differences among groups of treatment were analyzed by repeated measures ANOVA. Correlations were assessed by Spearman's rank correlation. Differences were considered significant at *P* < 0.05.

When considering clinical and analytical measures, missing data values were <1% either, at baseline and after 3 and 6 months of therapy.

Regarding molecular measures, missing data values at baseline were around 5%.

Because of a number of patients were not willing to donate blood samples after 6 months of therapy, and/or bleeding was not recommended by clinicians at this time, we achieved molecular data from approximately 75% of patients included in the study.

## Results

### MicroRNA Profile, Inflammatory, Oxidative Stress and NETosis-Derived Serum Parameters, Are Deregulated, Interrelated and Associated With the Clinical Profile of RA Patients

**Figure 1:**
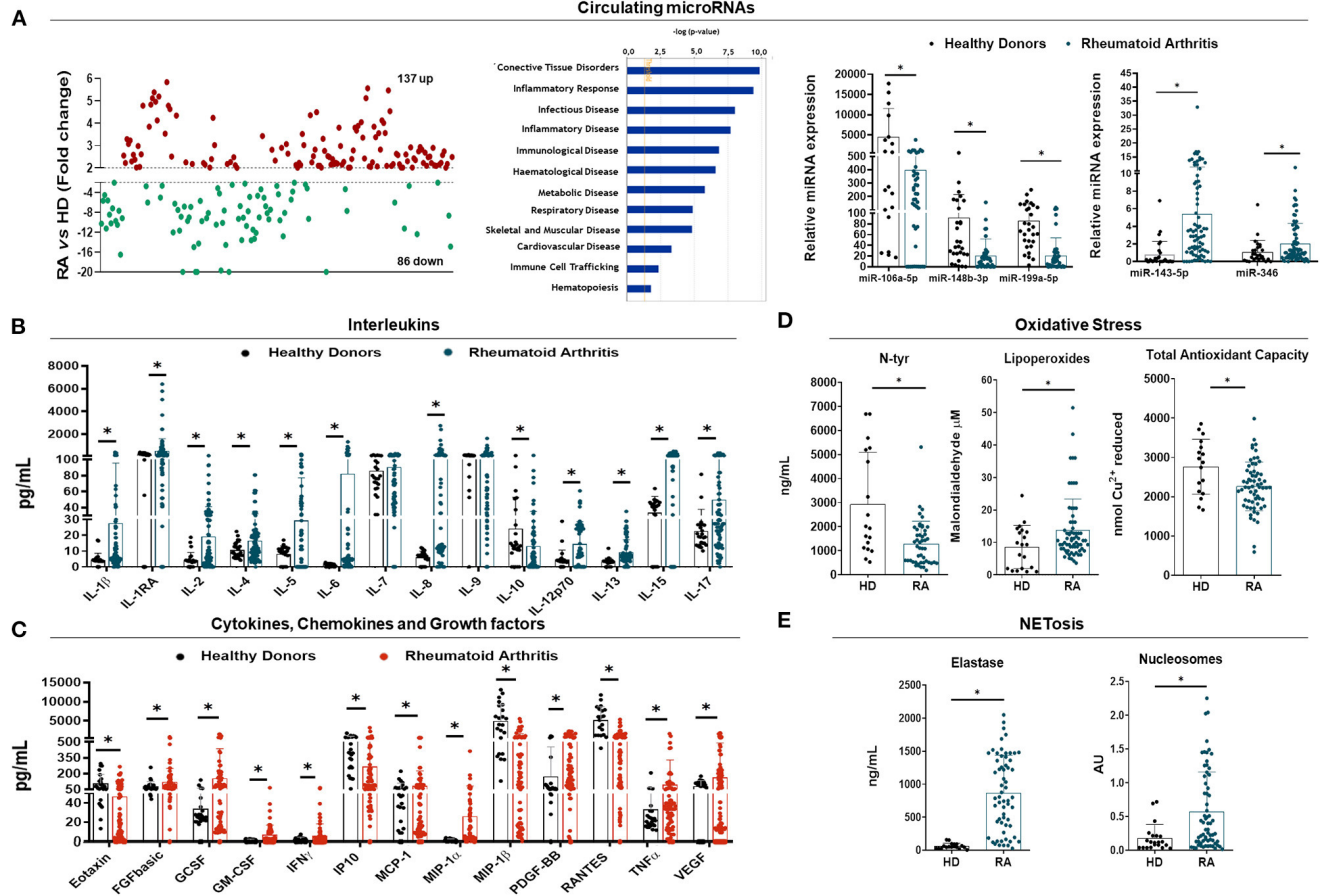
Molecular characterization of rheumatoid arthritis patients. **(A)** Whole circulating microRNA (miRNA) expression profile in plasma of Rheumatoid Arthritis (RA) patients (*n* = 6) and healthy donors (HDs) (*n* = 3) by HTG EdgeSeq Assay showing miRNAs up-regulated in red and miRNAs downregulated in green (Fold Change >2 or <-2) (Left panel). Functional classification of altered miRNAs according biological functions and diseases following Ingenuity Pathway Analysis (central panel). RT-PCR validation of selected miRNAs associated with the pathogenesis of RA (right panel). **(B)** Interleukin profile of RA patients and HDs in serum by Luminex Assay (Bio-Plex). **(C)** Cytokine, chemokine and growth factor profile of RA patients and HDs in serum by Luminex Assay (Bio-Plex). **(D)** Oxidative stress markers in serum of RA patients and HDs including Nitrotyrosine (N-Tyr), Lipoperoxides and Total Antioxidant Capacity (TAC). **(E)** NETosis-derived products in serum of RA patients and HDs including neutrophil elastase and nucleosome levels. Analyses were performed on the whole cohort of RA patients (*n* = 79) and HD (*n* = 29). *p < 0.05.

Whole miRNome profiling in serum samples identified altered levels of 223 miRNAs in RA patients before TNFi, when compared to HDs (cut-off: 2-fold change), of which 137 resulted to be upregulated and 86 downregulated (Figure 1A left panel).

By using the IPA software, the functional classification of these miRNAs revealed their association with clinical features of the RA physiopathology. Thus, the altered miRNAs signature in RA was enriched for biological processes such as connective tissue disorders, inflammatory response, infection, immunological, hematological, metabolic, respiratory and skeletal and muscular disorders, among others (Figure 1A central panel).

In order to validate the results in the whole cohort of RA patients and HDs, we performed an *in-silico* analysis that allowed the identification of a set of microRNAs as potential modulators of the expression of key targets involved in the pathology of RA. We identified a panel of 5 microRNAs including miR- 106a-5p, 143-5p, 148b-3p, 199a-5p, and 346 that showed potential targets molecules related to pro-inflammatory cytokines, chemokines, metalloproteinases, adhesion factors and critical immune receptors (BMP3, CCL-4, CCL-5, CCL-20, CXCL6, CXCL8, ITGA5, IL-6, IL6ST, IL-1RL1, MMP3, MMP13, TLR7, TNF, VCAM1, VEGFA, etc), along with a high number of molecules that regulate intracellular pathways associated with inflammatory and autoimmune processes (DKK-1,−2,−3, IKBKB, JAK1, MAPK14, MAP2K1, MAP3K5, NFKB1, NFßKBIA, PI3K3C2A, SOCS3, STAT3, WNT7, WNT2, WNT9B, etc.) (Supplementary Figure 2).

The expression of the 5 selected miRNAs was further analyzed by RT-PCR in the entire cohort, thus showing that the relative expression of all the selected circulating miRNAs was significantly altered in serum from RA patients when compared to HDs (*p* < 0.05) (Figure 1A right panel).

An inflammatory profile was also demonstrated in the serum of RA patients, including over-expression of a number of interleukins (IL-1β,−1RA,−2,−4,−5,−6,−8,−12,−13,−15 and−17) (Figure 1B), cytokines, chemokines and growth factors (Eotaxin, FGFbasic, GCSF, GM-CSF, IFNγ, MCP-1, MIP-1α, PDGF-BB, TNFα, and VEGF) (Figure 1C).

In addition, direct predicted miRNA-target interactions were identified between those deregulated miRNAs and several altered pro-inflammatory molecules (Supplementary Figure 3).

Increased NETs extrusion and enhanced oxidative status were also demonstrated by enlarged neutrophil cell-free elastase and Nucleosomes serum levels in RA patients (Figure 1E), along with increased levels of LPO and reduced N-Tyr and TAC (Figure 1D).

Correlation studies demonstrated a strong relationship among the levels of all the parameters evaluated, including inflammatory and oxidative stress markers, as well as with NETosis-derived products and microRNAs (Supplementary Figure 4).

### Unsupervised Cluster Analysis of the Integrated Serum Molecular Signatures Stratified RA Patients According to Their Disease Status

**Figure 2:**
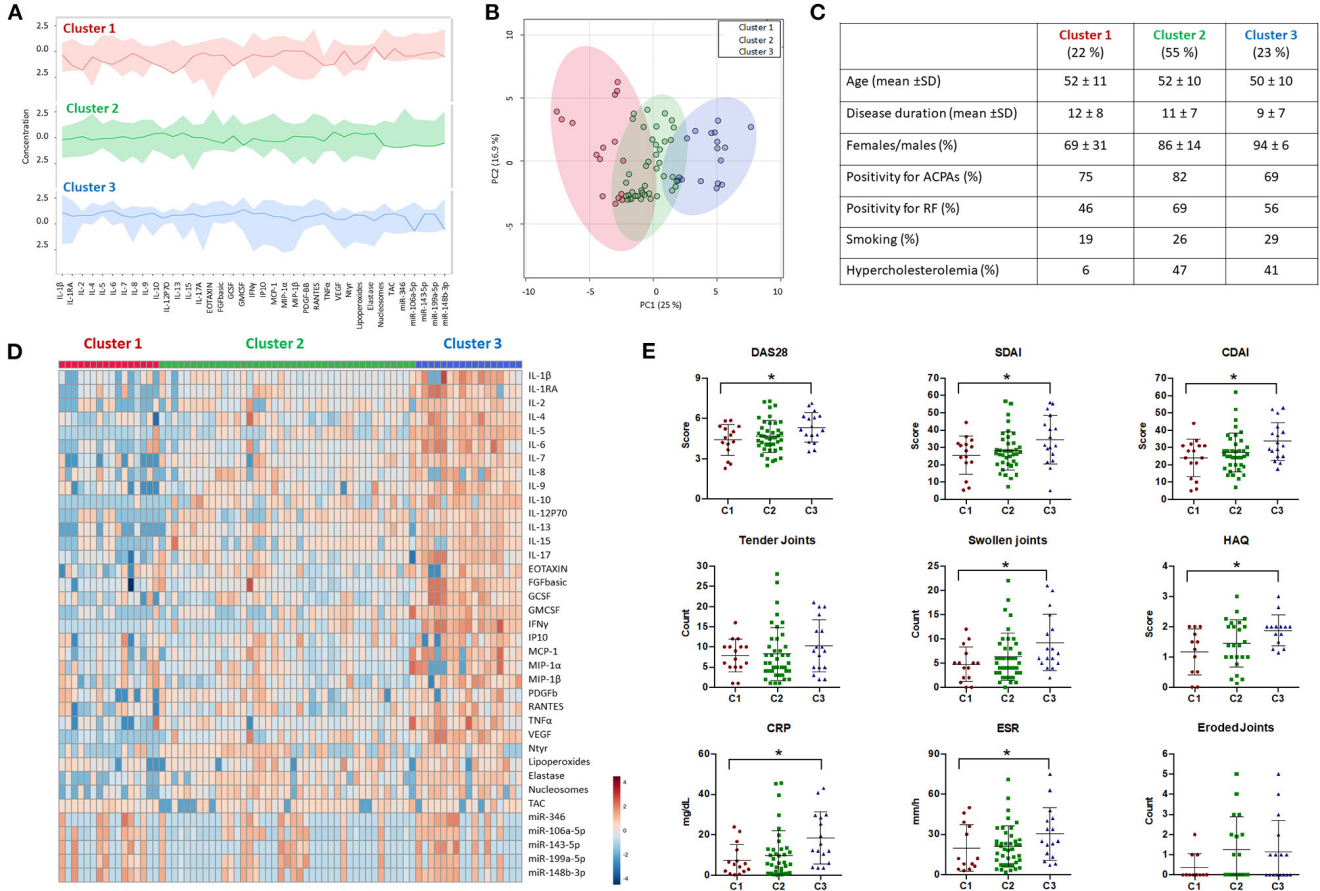
Cluster analysis of molecular features in rheumatoid arthritis patients. **(A)** Overview plot of the differential molecular profiles of Rheumatoid Arthritis (RA) patients (*n* = 74) using Self Organization Map (SOM) clustering analysis from MetaboAnalyst 4.0. The darker lines represent the median intensities of each cluster. **(B)** Principal component analysis (PCA) summarizing the differences in the molecular profile of each cluster. **(C)** Table of demographic and laboratory parameters of RA patients characterizing each cluster (Cluster 1 = 16; Cluster 2 = 41; Cluster 3 = 17). **(D)** Heatmap of the molecular profile of each cluster showing the normalized levels of all the biomolecules analyzed in RA patients. **(E)** Clinical features associated with each cluster including Disease Activity Score (DAS28), Simple Disease Activity index (SDAI), Clinical Disease Activity Index (CDAI), Tender and Swollen Joints, Health Assessment Questionnaire (HAQ), C-reactive protein (CRP), Erythrocyte Sedimentation Rate (ESR), and Bone Erosion. *p < 0.05.

By using self-organizing map (SOM) clustering analysis in the RA cohort, 3 clusters were distinguished, representing different serum molecular profile groups with respect to the circulating levels of inflammatory, oxidative and netotic mediators along with those of validated microRNAs (Figures 2A,C). Principal component analyses (PCA) confirmed a well-defined separation between these molecular clusters (Figure 2B). The clinical and laboratory profiles of each cluster were then evaluated (Figures 2C,E).

Briefly, cluster 1 (22% of the clustered cohort) was described on average by medium disease activity scores and low radiologic involvement, low prevalence of smokers and reduced prevalence of cardiovascular risk factors such as hypercholesterolemia.

On the contrary, cluster 3 (23% of the clustered cohort) comprised the patients with highest disease activity scores, along with a higher percentage of smokers and an enlarged prevalence of hypercholesterolemia.

Cluster 2 (55% of the clustered cohort) represented an intermediate clinical phenotype, though closer to cluster 3 in relation to disease activity scores, radiologic involvement, percentage of smokers and incidence of hypercholesterolemia.

No differences among clusters were found in relation to age, sex, positivity for autoantibodies, and disease duration.

Molecular analyses further recognized differential inflammatory, netotic, oxidative and miRNA profiles in RA patients' serum among clusters, on which patients belonging to clusters 1 and 3 displayed the most specific and distinctive expression patterns of interleukins, chemokines and growth factors, along with distinctive levels of oxidative stress markers such as lipoperoxides and N-Tyr, products of NETosis, including Elastase and Nucleosomes, and microRNAs (Figure 2D).

### Clinical Profile of RA Patients and Response to TNFi Therapy

At the start of the TNFi therapy all subjects showed medium-high disease activity, reflected by a mean DAS28 of 4.75 (2.05–7.5). A 74% of patients took non-steroidal anti-inflammatory drugs (NSAIDS) daily, and 92% received steroid treatment (range 2.5–30 mg/d prednisone). Methotrexate alone or in combination with other DMARD was further administered in 61% of subjects.

**Figure 3:**
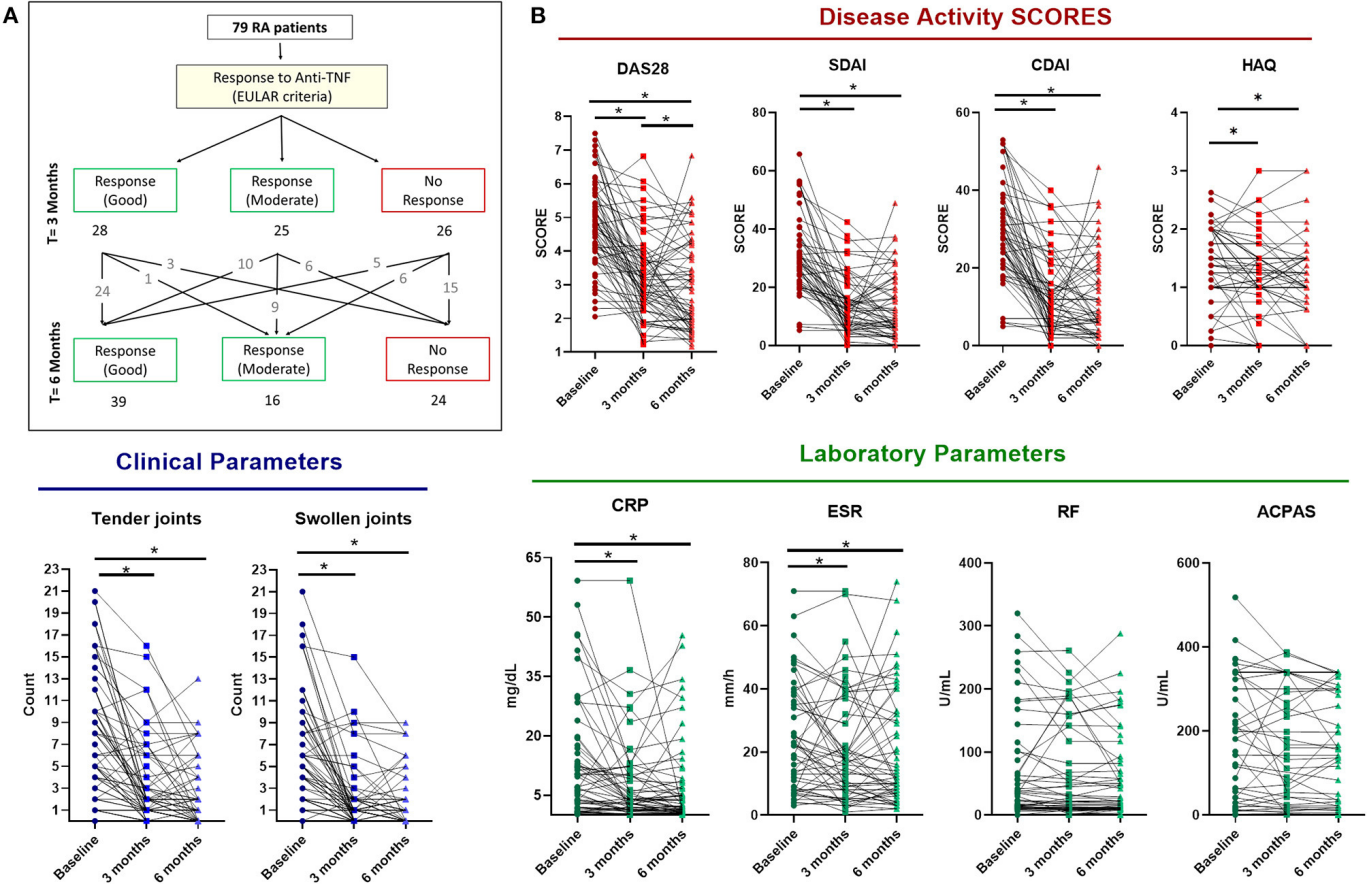
Clinical response to anti-TNF therapy in rheumatoid arthritis patients. **(A)** Flow diagram representing the clinical response at 3 and 6 months of anti-TNF therapy following EULAR criteria. **(B)** RA patients' (*n* = 79) changes in clinical features at 3 and 6 months of anti-TNF therapy including Disease Activity Score (DAS28), Simple Disease Activity index (SDAI), Clinical Disease Activity Index (CDAI), Tender and Swollen Joints, Health Assesment Questionnaire (HAQ), C-reactive protein (CRP), erythrocyte Sedimentation Rate (ESR), Rheumatoid Factor (RF) and Anticitrullinated protein antibodies (ACPAS). *p < 0.05.

According to DAS28 response criteria, at 3 months of treatment, 28 (35%), 25 (32%), and 26 (33%) RA patients showed good, moderate, and no response to TNFi therapy, respectively (Figure 3A). All the clinical parameters evaluated, including DAS28, SDAI and CDAI scores, along with HAQ, number of swollen and tender joints improved significantly. In addition, acute phase reactants were also reduced (Figure 3B). Interestingly, most of good responders at 3 months remained responsive to therapy at 6 months. Besides, a significant number of moderate responders shifted to good responders in relation to those that remained as moderate or changed to non-responders (Figure 3A).

All five biological agents had a favorable influence on the evolution of those parameters, so that changes were not influenced by treatment with monoclonal antibodies nor with soluble receptor (Supplementary Figure 5A). Considering the clinical response more consolidated at 6 months of treatment, we selected this time after starting therapy to assess serum molecular changes and to search for potential biomarkers as predictors of response.

### TNFi Changes on Serum Molecular Profile of RA Patients Were Specific of the Cluster Evaluated and Associated With the Clinical Response

**Figure 4:**
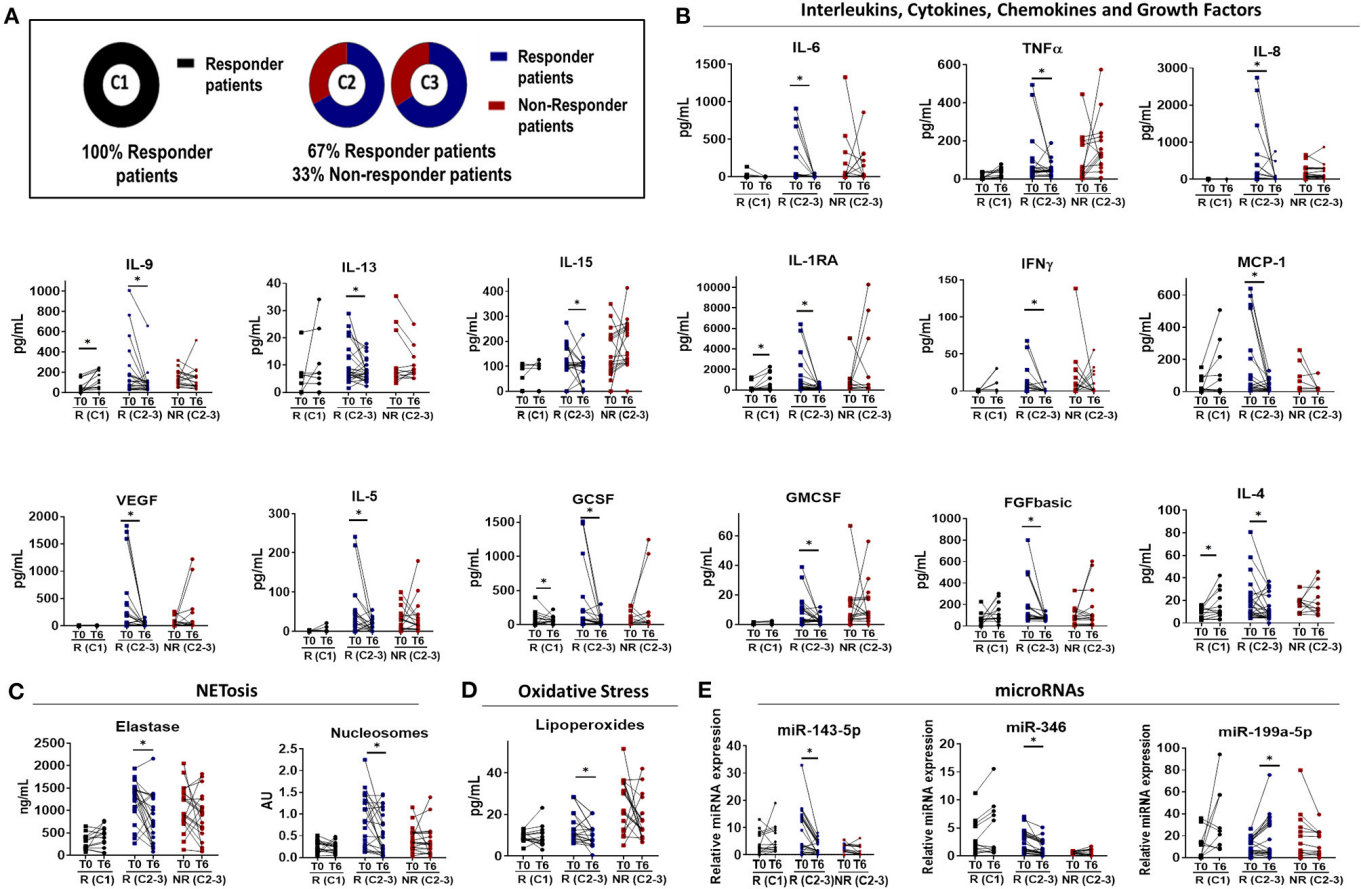
Molecular response to anti-TNF therapy in rheumatoid arthritis patients. **(A)** Diagram showing the distribution of EULAR responder and non-responder patients among the different molecular clusters that characterized Rheumatoid Arthritis (RA) patient at baseline. Cluster 1 was characterized by responder patients while non-responder patients were identified only in cluster 2 and 3. **(B–E)** Individual changes in the level of biomolecules related to inflammation **(B)**, NETosis **(C)**, oxidative stress **(D)**, and microRNAs **(E)** before and after 6 months of Anti-TNF-therapy between responders and non-responder patients. *p < 0.05. R (C1), Responder patients of Cluster 1 (*n* = 16); R (C2-3), Responder patients of Cluster 2 and 3 (*n* = *25*); NR (C2-3), Non-Responder patients of Cluster 2 and 3 (*n* = *17*).

According to EULAR response criteria, all patients belonging to cluster 1 showed clinical response after 6 months of therapy. In clusters 2 and 3, 67% of patients were responders and a 33% of them were non-responders (Figure 4A). Thus, considering the similarity of these clusters concerning both, the clinical and molecular profiles and the response to treatment, and in order to identify molecular mechanisms of non-response, we decided to evaluate jointly the molecular changes occurred in these clusters after TNFi therapy.

As a general feature, we identified two main distinctive molecular profiles among RA patients responders to TNFi, involving, on one hand, low-medium baseline levels of inflammatory, oxidative and netotic mediators in those belonging to cluster 1, and on the other hand, high baseline levels of these parameters in responders belonging to the other two clusters.

Accordingly, in cluster 1 we observed few or no changes in the levels of these parameters after 6 months of therapy. On the contrary, the clinical response to TNFi in clusters 2 and 3 was found linked to a significant reduction in levels of a number of inflammatory mediators, oxidative stress markers, and products of NETosis, along with the restoration in the levels of microRNAs. These changes were not observed in patients that did not display clinical response to therapy (Figure 4).

**Figure 5:**
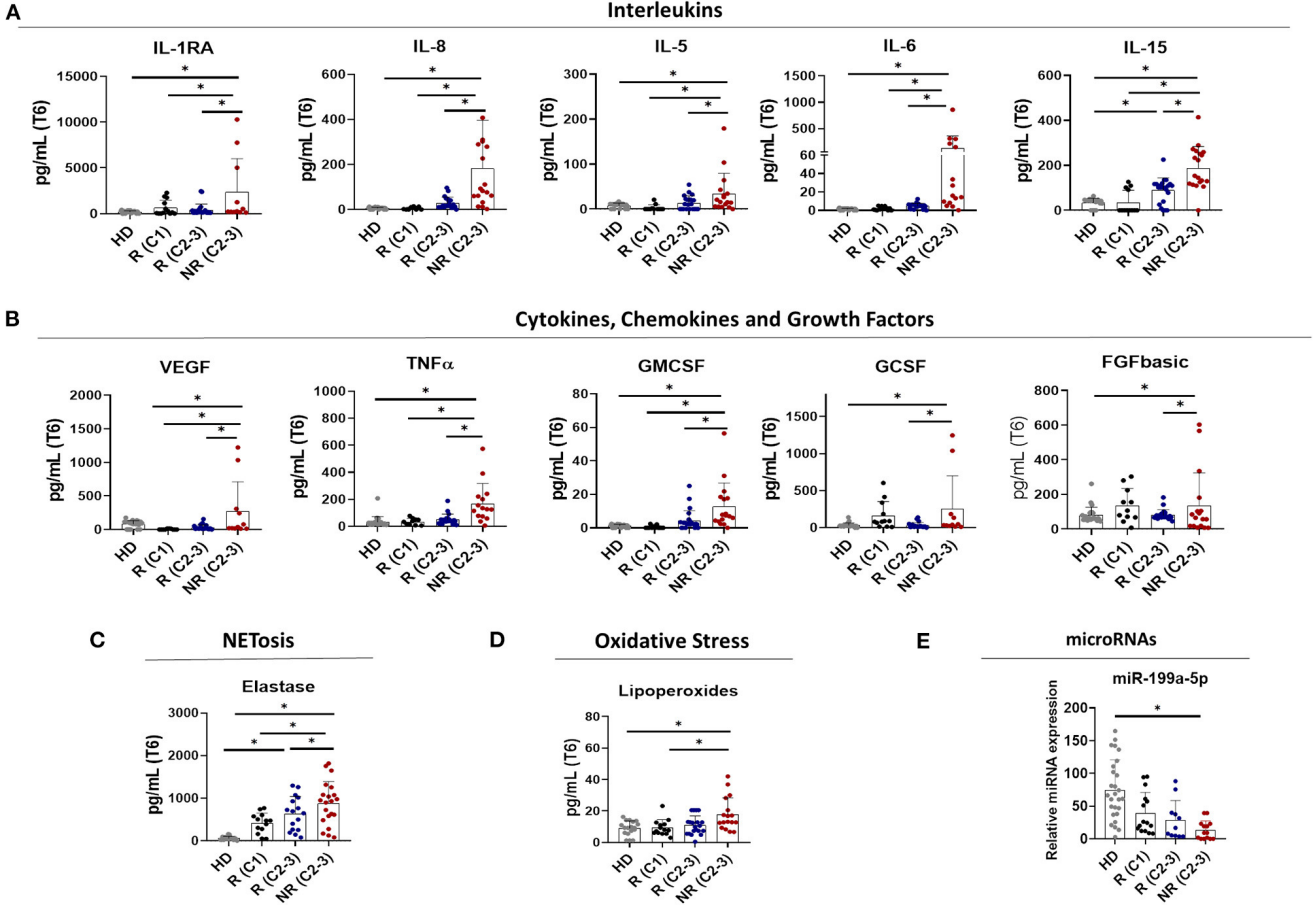
Increased levels of altered biomolecules in non-responder patients to anti-TNF therapy after 6 months of treatment. Level of Interleukins **(A)**, Cytokines, Chemokines and Growth Factors **(B)**, NETosis-derived products **(C)**, oxidative stress markers **(D)**, and circulating microRNAs **(E)** in the plasma of Healthy donors (HDs) and responder and non-responder patients after 6 months of Anti-TNF therapy. *p< 0.05. R (C1), Responder patients of Cluster 1 (*n* = 16); R (C2-3), Responder patients of Cluster 2 and 3 (*n* = *25*); NR (C2-3), Non-Responder patients of Cluster 2 and 3 (*n* = *17*).

Moreover, we observed that in responders patients to TNFi therapy, the levels of most of these parameters reached the ranks found in healthy donors after 6 months of therapy, while in non-responders, these parameters remained significantly elevated (Figure 5).

It must be noted that changes observed in molecular parameters were similar among patients treated with either, monoclonal anti-TNF antibodies or soluble receptor, independently of both, the cluster on which patients were included and the clinical response to therapy (Supplementary Figure 5B).

**Figure 6:**
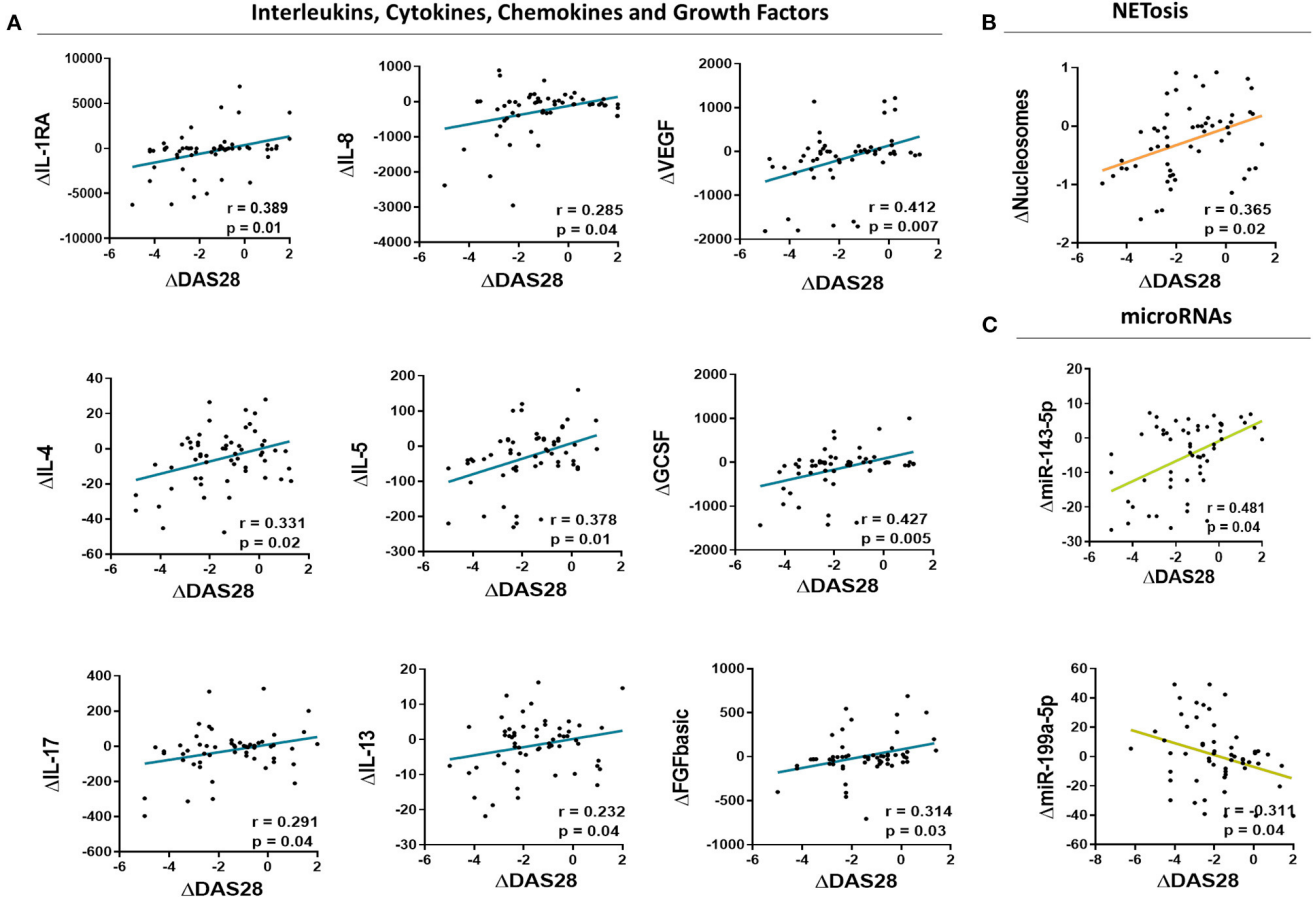
Correlations among changes in the levels of altered biomolecules and the clinical response induced by anti-TNF therapy in rheumatoid arthritis patients. Correlation analysis among changes in the levels of inflammatory mediators **(A)**, NETosis markers **(B)** and microRNAs **(C)** in the serum of Rheumatoid Arthritis patients after 6 months of anti-TNF Therapy and changes in the disease activity score (DAS28).

In support for these results, we further observed a significant positive correlation among the changes arisen in the levels of these molecules and the improvement of the disease activity, identified by DAS28 score (Figure 6).

### Machine Learning Algorithms Allowed the Identification of Potential Predictors of TNFi Response

By using machine-learning algorithms such as logistic regression models, we searched for potential predictors of TNFi response based on clinical and molecular profiles of RA patients before starting therapy.

**Figure 7:**
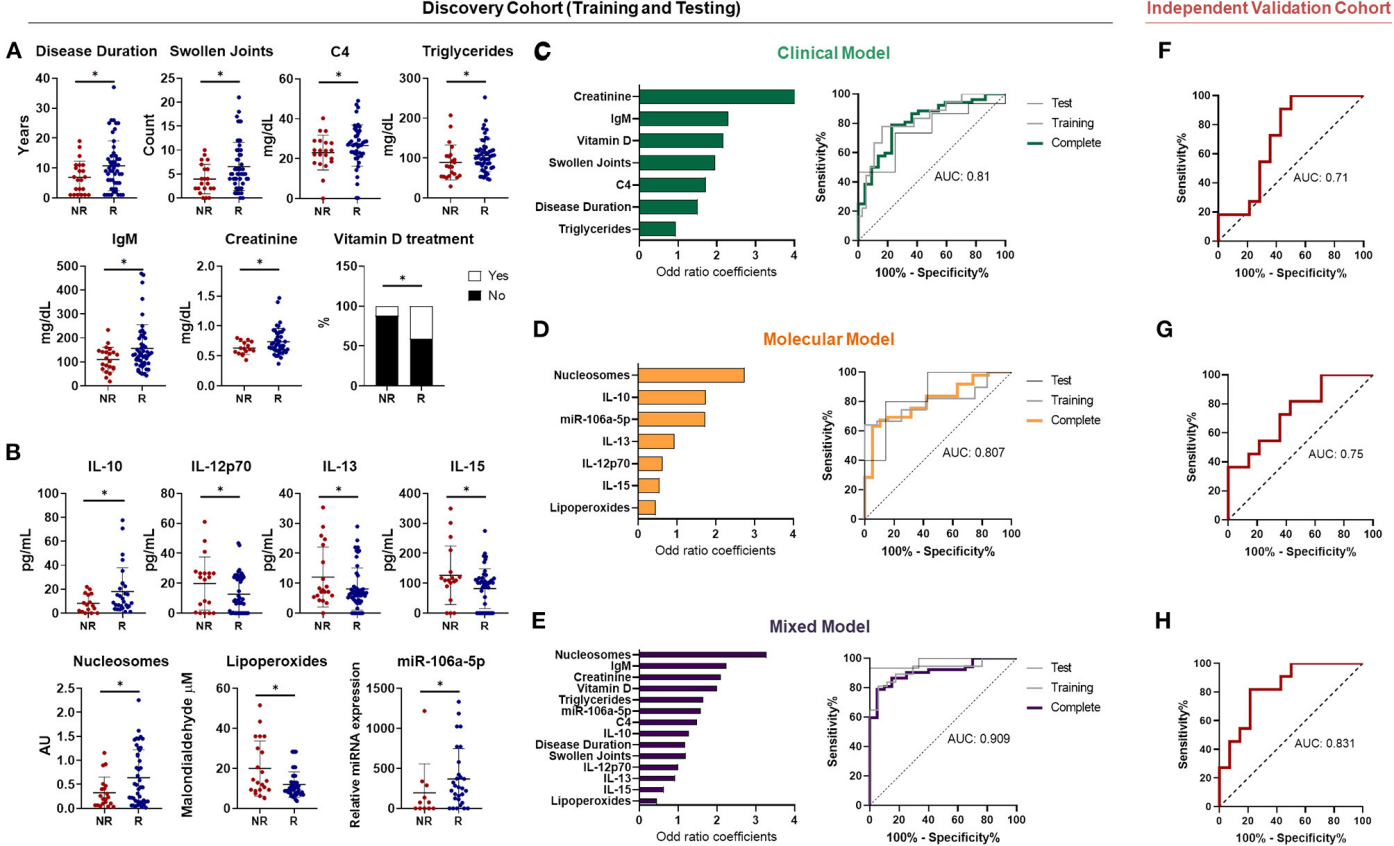
Biomarkers predictors of anti-TNF response in rheumatoid arthritis by using machine learning. **(A)** Baseline clinical variables associated with the EULAR clinical response to Anti-TNF in Rheumatoid Arthritis (RA) patients after 6 months (*n* = 74, including 52 R and 22 NR). **(B)** Baseline molecular variables associated with the EULAR clinical response in RA patients after 6 months. **(C)** ROC curve of the machine learning model predictor of clinical response (left panel) using only clinical variables and their individual contribution represented by the odd ratio coefficients (right panel). **(D)** ROC curve of the machine learning model predictor of clinical response (left panel) using only molecular variables and their individual contribution represented by the odd ratio coefficients (right panel). **(E)** ROC curve of the machine learning model predictor of clinical response (left panel) using the best combination of clinical and molecular variables and their individual contribution represented by the odd ratio coefficients (right panel). **(F)** ROC curve of the machine learning model predictor of clinical response in an independent validation cohort. **(G)** ROC curve of the machine learning model predictor of molecular response in an independent validation cohort. **(H)** ROC curve of the machine learning model predictor using both clinical and molecular variables. (validation cohort: *n* = 25, including 14 R and 11 NR). *p < 0.05. R, responders; NR, non responders; AUC, area under the curve.

Firstly, we identified the clinical and molecular parameters that significantly distinguished among responder and non-responder patients, based on EULAR criteria (Figures 7A,B). Then, the identified parameters were split in clinical and molecular groups and logistic models were performed.

Among clinical groups, logistic model identified as good response factors, higher levels of creatinine, complement C4, total IgM, number of swollen joints, longer disease duration and complementary therapy with vitamin D, showed by odd ratio (OR) coefficients > 1. ROC curve analyses demonstrated that the combined model of these parameters identified responder patients with high accuracy (AUC = 0.81) (Figure 7C).

Among molecular parameters, logistic model identified as good response factors, high levels of nucleosomes, IL-10, miR106a-5p and IL-13, (OR > 1). Yet, high levels of lipoperoxides, IL-15 and IL-12p70 were predictors of non response (OR < 1). Likewise, ROC curve analysis with the combined model of molecular variables identified responder patients with similar accuracy (AUC = 0.807) (Figure 7D).

Interestingly, in the mixed model, combining clinical and molecular features, ROC curve showed better discriminative capacity than each single model (AUC = 0.909), thus supporting the relevance of combining both, clinical and molecular features to allow an accurate prediction of TNFi response (Figure 7E).

Moreover, similar prediction patterns were found when compared the treatments with soluble receptor vs. monoclonal antibodies. In fact, predictive models that mixed clinical and molecular features fit well for both types of TNFi, displaying areas under the curve close to 0.9 (Supplementary Figure 6). This data reinforces the potential clinical utility of this mixed model.

Likewise, when we evaluated the performance of these models in an independent cohort of 25 patients (invonving 14 responders and 11 non-responder patients to TNFi), we could validate their capacity to predict the response to TNFi before therapy. Thus, the mixed model integrating clinical and molecular features predicted the response with an AUC of 0.83, which was significantly higher compared to separated clinical and molecular models as in the case of the discovery cohort (Figures 7F–H).

## Discussion

Continuous updating of the knowledge on molecular processes associated to the pathogenesis of RA, and on the specific effects of bDMARDs in the correction of their dysregulation, are essential in the early and correct approach to the treatment of this complex autoimmune disorder. RA is a dissimilar disease, involving multiple clinical manifestations and pathogenic mechanisms among individuals with the same diagnosis and/or throughout different disease stages. These traits support the complexity of the disease and the involvement of numerous factors in the trigger and the evolution of RA (17).

The present longitudinal study has elucidated the effects of TNFi treatment at the molecular level. In addition, a comprehensive analysis that combines data from different molecular categories and detailed clinical parameters has allowed a better understanding of molecular systems linked to disease severity effects of drugs treatment. Therefore, we demonstrated that, in parallel to the clinical response, TNFi promoted the re-establishment in the levels of circulating inflammatory and oxidative stress mediators, a significant reduction of NETosis-derived bioproducts and a substantial reversal of altered miRNAs.

It has been suggested that the diversity of biological processes underlying the pathogenesis of RA implies that their clinical phenotypes represent jointly altered pathways rather than exemplify the outcomes of single altered entities. Accordingly, different biological therapies have demonstrated numerous beneficial molecular effects that modulate pathological processes of the disease in RA (18). In the present study we identified a number of molecular alterations that develops jointly in these patients and are intimately associated to distinctive clinical phenotypes.

Firstly, clustering analysis based on molecular profiles before TNFi therapy, allowed the unsupervised division of three groups of RA patients, showing distinctive clinical phenotypes, further linked to the effectiveness of TNFi treatment. Thus, cluster 1 comprised patients 100% responders to therapy, who displayed a less prominent inflammatory status at baseline. On the contrary, on clusters 2 and 3, RA responders' patients to TNFi therapy were characterized by a high inflammatory status that paralleled a high disease activity. Patients from cluster 1 did not show changes in the molecular panel evaluated. However, the fact that all of them achieved a clinical response suggests that changes in other inflammatory mediators might be responsible for the therapy effectiveness. In these patients from cluster 2 and 3, therapy promoted a clear inflammatory response, involving the downregulation of cytokines, chemokines, growth factors, NETosis bioproducts and oxidative stress molecules. Quite the reverse, in non-responder patients belonging to these two clusters, the high levels found of inflammatory mediators and other altered metabolites were not reduced by TNFi, remaining increased after 6 months of therapy. Similar results have been published in previous works, including ours (19). Although core mechanisms remain to be clarified, this is the first study that identifies two sets of patients with distinctive molecular profiles at baseline that share a good response to TNFi therapy, underlying a heterogeneity in these patients that most probably derives of numerous clinical, genetic and environmental factors awaiting to be clarified.

In addition, our data strengths the relevance of integrating molecular and clinical studies in these patients, in order to identify potential predictors of treatment response. Hence, by using machine-learning algorithms (i.e., logistic regression models) we searched for potential predictors of TNFi response based on clinical and molecular profiles of RA patients. Our results identified signatures involving both clinical and molecular parameters that might predict the response to TNFi, which was further independent of the TNF inhibitor used, either monoclonal antibody or soluble receptor. Thus, several inflammatory cytokines, along with bioproducts of NETosis, oxidative stress and microRNAs, whose altered levels have been previously demonstrated to be altered in RA patients and modified by bDMARDs, were predictors of response to TNFi. Consistently, among clinical parameters, longer disease evolution, high number of swollen joints, and increased serum levels of creatinine, triglycerides, complement C4 and IgM, along with the concomitant treatment with vitamin D, were associated to the response to TNFi after 6 months of treatment.

Preceding studies demonstrated that these parameters were linked to the altered autoimmune and inflammatory status of RA patients (i.e., inflamed joints, elevated IgM and complement C4) (20, 21), and/or were indicative of an active metabolic status (i.e., high creatinine) (22). Moreover, available evidence indicates that high inflammation interferes with lipid metabolism, so that hyperlipidemia is frequently associated to the adverse clinical outcome of the disease, and good control of the chronic inflammatory state may positively influence the lipid profile (23).

Additionally, several studies have shown that supplementary vitamin D can effectively control the DAS28, TJC and ESR levels in RA patients, mainly due to its anti-inflammatory properties (24). Correspondingly, in our hands, patients having a supplemental vitamin D treatment showed a better response to TNFi. Similar results have been also shown in other inflammatory diseases such Inflammatory Bowel Disease where higher levels of Vitamin D are associated with greater odds of remission with TNFi (25).

Hence, in our cohort, signatures involving clinical and molecular parameters associated to a more significantly altered inflammatory and metabolic status at baseline, and a concomitant therapy with a compound with anti-inflammatory properties -such as vitamin D-, seems to identify those patients who could benefit more from TNFi treatment.

Machine learning is a new field gaining attention in Rheumatology (26). Thus, two recent studies have shown their potential to predict TNFi response using clinical or molecular data. Guan et al., showed that large collection of clinical data at baseline along with a Gaussian process regression model correctly classified 78% of responder patients (27). In line with this, Tao et al. showed the capacity of gene expression and DNA methylation to predict TNFi response in RA using random forest algorithms with an accuracy of 85% (28).

In our study, we demonstrated for the first time that the combination of molecular and clinical data using machine learning exhibited a greater capacity to predict the clinical response to TNFi therapy in RA patients than each one separately. This finding could pave the way for the development of larger and independent validation studies aimed to achieve a precision medicine model to predict TNFi in RA and related diseases. Likewise, future analysis of predictors of therapy response using other biological drugs with different mechanism of action (Anti-CD20, Anti-IL6, JAK-inhibitors etc.) will be also required for the development of a wide approach of personalized medicine in RA patients in which all the available drugs are included.

Limitations of the study: Firstly, since we did not perform a complete serum profile involving inflammatory mediators, oxidative stress markers, NETotic bioproducts and miRNAs, we cannot exclude the complementary role of other circulating biomolecules, -including non-evaluated inflammatory molecules or miRNAs, among others- in the response to treatment. In addition, due to the clinical heterogeneity of RA patients and the relatively small number of patients analyzed in this study, data must be confirmed in larger cohorts. Moreover, specific analyses on the mechanisms underlying the altered expression of these molecules after TNFi therapy, as well as the identification of cellular sources of these circulating biomolecules are still required.

Taken together, our overall data suggest that:

1. RA patients undergoing anti-TNF-therapy conform distinctive clusters based on altered molecular profiles, which are directly linked to their clinical status at baseline.
2. Clinical effectiveness of anti-TNF therapy was divergent among these molecular clusters and associated with a specific modulation of the inflammatory response, the reestablishment of the altered oxidative status, the reduction of NETosis, and the reversion of related altered miRNAs.
3. Through a systematic and comprehensive study design including discovery and validation phases, we have developed an integrative analysis of the clinical and molecular profiles of RA patients using machine learning, which allowed the identification of novel signatures as potential predictors of therapeutic response to TNFi therapy.

Our overall data pave the way for the development of large prospective and validation studies, needed to achieve a personalized medicine approach for RA patients.

## Data Availability Statement

The original contributions presented in the study are included in the article/Supplementary Material, further inquiries can be directed to the corresponding author/s.

## Ethics Statement

The studies involving human participants were reviewed and approved by Ethics Committee of Reina Sofia Hospital. The patients/participants provided their written informed consent to participate in this study.

## Author Contributions

ML-T, CP-S, AE-C, EC-E, and CL-P formed the hypothesis, directed and coordinated the project, designed the experiments, analyzed the data and wrote the manuscript. DR-V, RO-C, MR-G, CR-E, JP-V, MR-M, CD, CR-B, AF-N, NM-V, JM, JU-M, MT-C, and MA followed up with patients and contributed useful discussion and suggestions. NB, MA-A, AP-T, and IA developed the *in vivo* assays and solved technical problems. JM-S CL-M, and PF were involved in statistical analysis and discussing related results. All authors contributed to the article and approved the submitted version.

## Conflict of Interest

The authors declare that the research was conducted in the absence of any commercial or financial relationships that could be construed as a potential conflict of interest.

## References

[1] McInnes IB Schett G. The pathogenesis of rheumatoid arthritis. N Engl J Med. (2011) 365:2205–19. 10.1056/NEJMra1004965 22150039

[2] Chen Z Bozec A Ramming A Schett G. Anti-inflammatory and immune regulatory cytokines in rheumatoid arthritis. Nat Rev Rheumatol. (2019) 15:9–17. 10.1038/s41584-018-0109-2 30341437

[3] Johnson KJ Sanchez HN Schoenbrunner N. Defining response to TNF-inhibitors in rheumatoid arthritis: the negative impact of anti-TNF cycling and the need for a personalized medicine approach to identify primary non-responders. Clin Rheumatol. (2019) 38:2967–976. 10.1007/s10067-019-04684-1 31520227

[4] Katchamart W Johnson S Lin HJL Phumethum V Salliot C Bombardier C. Predictors for remission in rheumatoid arthritis patients: a systematic review. Arthrit Care Res. (2010) 62:1128–43. 10.1002/acr.20188 20235210

[5] Callaghan C Boyter A Mullen A McRorie E. Biological therapy for rheumatoid arthritis: is personalised medicine possible? Eur J Hosp Pharm. (2014) 21:229–37. 10.1136/ejhpharm-2013-000386

[6] Vastesaeger N Kutzbach AG Amital H Pavelka K Lazaro MA Moots RJ. Prediction of remission and low disease activity in disease-modifying anti-rheumatic drug-refractory patients with rheumatoid arthritis treated with golimumab. Rheumatology. (2016) 55:1466–76. 10.1093/rheumatology/kew179 27114562 PMC4957672

[7] Ganhão S Bernardes M Lucas R Fonseca J Rosa-Gonçalves D Madeira N. Remission and low disease activity matrix tools: results in real-world rheumatoid arthritis patients under anti-tnfa therapy. Acta Reumatol Portuguesa. (2019) 45:245–52. 33420771

[8] Smallwood MJ Nissim A Knight AR Whiteman M Haigh R Winyard PG. Oxidative stress in autoimmune rheumatic diseases. Free Radic Biol Med. (2018) 125:3–14. 10.1016/j.freeradbiomed.2018.05.086 29859343

[9] Grayson PC Kaplan MJ. At the bench: neutrophil extracellular traps (NETs) highlight novel aspects of innate immune system involvement in autoimmune diseases. J Leukoc Biol. (2016) 99:253–64. 10.1189/jlb.5BT0615-247R 26432901 PMC4718195

[10] Pérez-Sánchez C Ruiz-Limón P Aguirre MA Jiménez-Gómez Y Arias-de la Rosa I Ábalos-Aguilera MC. Diagnostic potential of NETosis-derived products for disease activity, atherosclerosis and therapeutic effectiveness in rheumatoid arthritis patients. J Autoimmun. (2017) 82:31–40. 10.1016/j.jaut.2017.04.007 28465139

[11] Perez-Sanchez C Cecchi I Barbarroja N Patino-Trives AM Luque-Tevar M Perez-Sanchez L. Early restoration of immune and vascular phenotypes in systemic lupus erythematosus and rheumatoid arthritis patients after B cell depletion. J Cell Mol Med. (2019) 23:6308–18. 10.1111/jcmm.14517 31347786 PMC6714224

[12] Ruiz-Limón P Ortega R Arias de la Rosa I Ábalos-Aguilera MDC Pérez-Sánchez C Jiménez-Gómez Y. Tocilizumab improves the proatherothrombotic profile of rheumatoid arthritis patients modulating endothelial dysfunction, NETosis, and inflammation. Transl Res. (2017) 183:87–103. 10.1016/j.trsl.2016.12.003 28027930

[13] Sanchez-Pinto LN Luo Y Churpek MM. Big data and data science in critical care. Chest. (2018) 154:1239–48. 10.1016/j.chest.2018.04.037 29752973 PMC6224705

[14] Aletaha D Neogi T Silman AJ Funovits J Felson DT Bingham CO 3rd. 2010 rheumatoid arthritis classification criteria: an American College of Rheumatology/European League Against Rheumatism collaborative initiative. Arthritis Rheum. (2010) 62:2569–81. 10.1002/art.27584 20872595

[15] Hosmer DW Jr Lemeshow S Sturdivant RX. Applied Logistic Regression. New Jersey, NJ: John Wiley & Sons (2013).

[16] Pedregosa F Varoquaux G Gramfort A Michel V Thirion B Grisel O. Scikit-learn: machine learning in python. J Mach Learn Res. (2011) 12:2825–30.

[17] Smolen JS Aletaha D Barton A Burmester GR Emery P Firestein GS. Rheumatoid arthritis. Nat Rev Dis Primers. (2018) 4:18001. 10.1038/nrdp.2018.1 29417936

[18] Conigliaro P Triggianese P De Martino E Fonti GL Chimenti MS Sunzini F. Challenges in the treatment of rheumatoid arthritis. Autoimmun Rev. (2019) 18:706–13. 10.1016/j.autrev.2019.05.007 31059844

[19] Castro-Villegas C Pérez-Sánchez C Escudero A Filipescu I Verdu M Ruiz-Limón P. Circulating miRNAs as potential biomarkers of therapy effectiveness in rheumatoid arthritis patients treated with anti-TNFalpha. Arthritis Res Ther. (2015) 17:49. 10.1186/s13075-015-0555-z 25860297 PMC4377058

[20] Lin Y-J Anzaghe M Schülke S. Update on the pathomechanism, diagnosis, and treatment options for rheumatoid arthritis. Cells. (2020) 9:880. 10.3390/cells9040880 32260219 PMC7226834

[21] Vignesh P Rawat A Sharma M Singh S. Complement in autoimmune diseases. Clin Chim Acta. (2017) 465:123–30. 10.1016/j.cca.2016.12.017 28040558

[22] Kapoor SR Filer A Fitzpatrick MA Fisher BA Taylor PC Buckley CD. Metabolic profiling predicts response to anti–tumor necrosis factor α therapy in patients with rheumatoid arthritis. Arthritis Rheum. (2013) 65:1448–56. 10.1002/art.37921 23460124 PMC3715109

[23] Cacciapaglia F Anelli MG Rinaldi A Serafino L Covelli M Scioscia C. Lipid profile of rheumatoid arthritis patients treated with anti-tumor necrosis factor-alpha drugs changes according to disease activity and predicts clinical response. Drug Dev Res. (2014) 75:S77–80. 10.1002/ddr.21203 25381986

[24] Jeffery LE Raza K Hewison M. Vitamin D in rheumatoid arthritis—towards clinical application. Nat Rev Rheumatol. (2016) 12:201–10. 10.1038/nrrheum.2015.140 26481434

[25] Winter RW Collins E Cao B Carrellas M Crowell AM Korzenik JR. Higher 25-hydroxyvitamin D levels are associated with greater odds of remission with anti-tumour necrosis factor-α medications among patients with inflammatory bowel diseases. Aliment Pharmacol Ther. (2017) 45:653–9. 10.1111/apt.13936 28074487 PMC5290123

[26] Pandit A Radstake TR. Machine learning in rheumatology approaches the clinic. Nat Rev Rheumatol. (2020) 16:69–70. 10.1038/s41584-019-0361-0 31908355

[27] Guan Y Zhang H Quang D Wang Z Parker SC Pappas DA. Machine learning to predict anti–tumor necrosis factor drug responses of rheumatoid arthritis patients by integrating clinical and genetic markers. Arthritis Rheumatol. (2019) 71:1987–96. 10.1002/art.41056 31342661

[28] Tao W Concepcion AN Vianen M Marijnissen AC Lafeber FP Radstake TR. Multi-omics and machine learning accurately predicts clinical response to adalimumab and etanercept therapy in patients with rheumatoid arthritis. Arthritis Rheumatol. (2020). 10.1002/art.41516. [Epub ahead of print]. PMC7898388 32909363

