# Supplementary material for: Integrative Clinical, Molecular, and Computational Analysis Identify Novel Biomarkers and Differential Profiles of Anti-TNF Response in Rheumatoid Arthritis
Source: Front Immunol. 2021 Mar 23;12:631662. doi: 10.3389/fimmu.2021.631662 (PMC8022208; doi:10.3389/fimmu.2021.631662)
Supplement: Supplementary file 1 [file Data_Sheet_1.doc]

**Supplementary Information**

**Supplementary Figures**

**
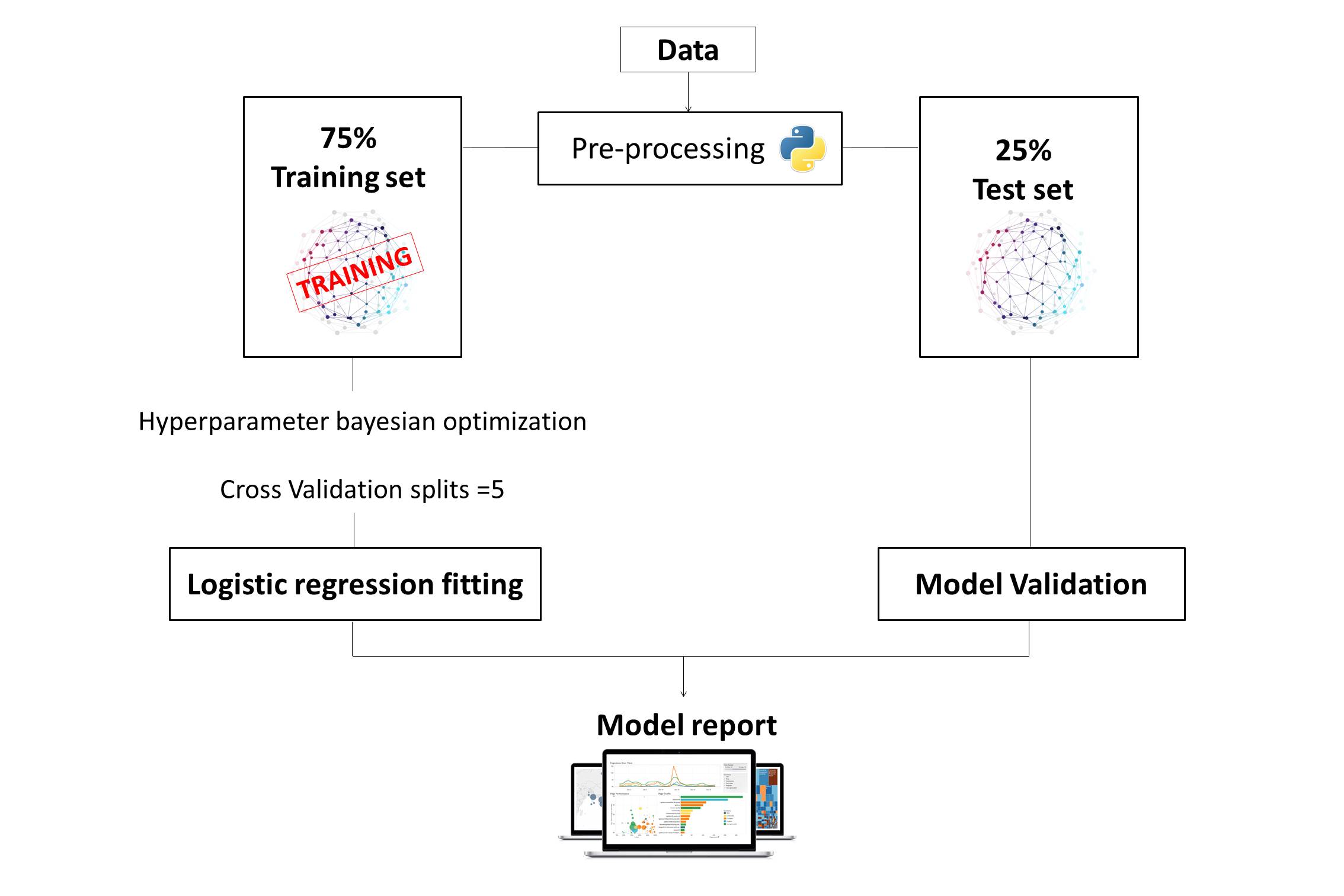
**

**Supplemental Figure 1. Overview of the Machine Learning approach.** Flow chart showing the machine learning methodology used for predicting clinical response to Anti-TNF therapy after 6 months of treatment in Rheumatoid Arthritis patients. Models were generated by using logistic regression analysis using two datasets including the training (75%) and the test set (25%).


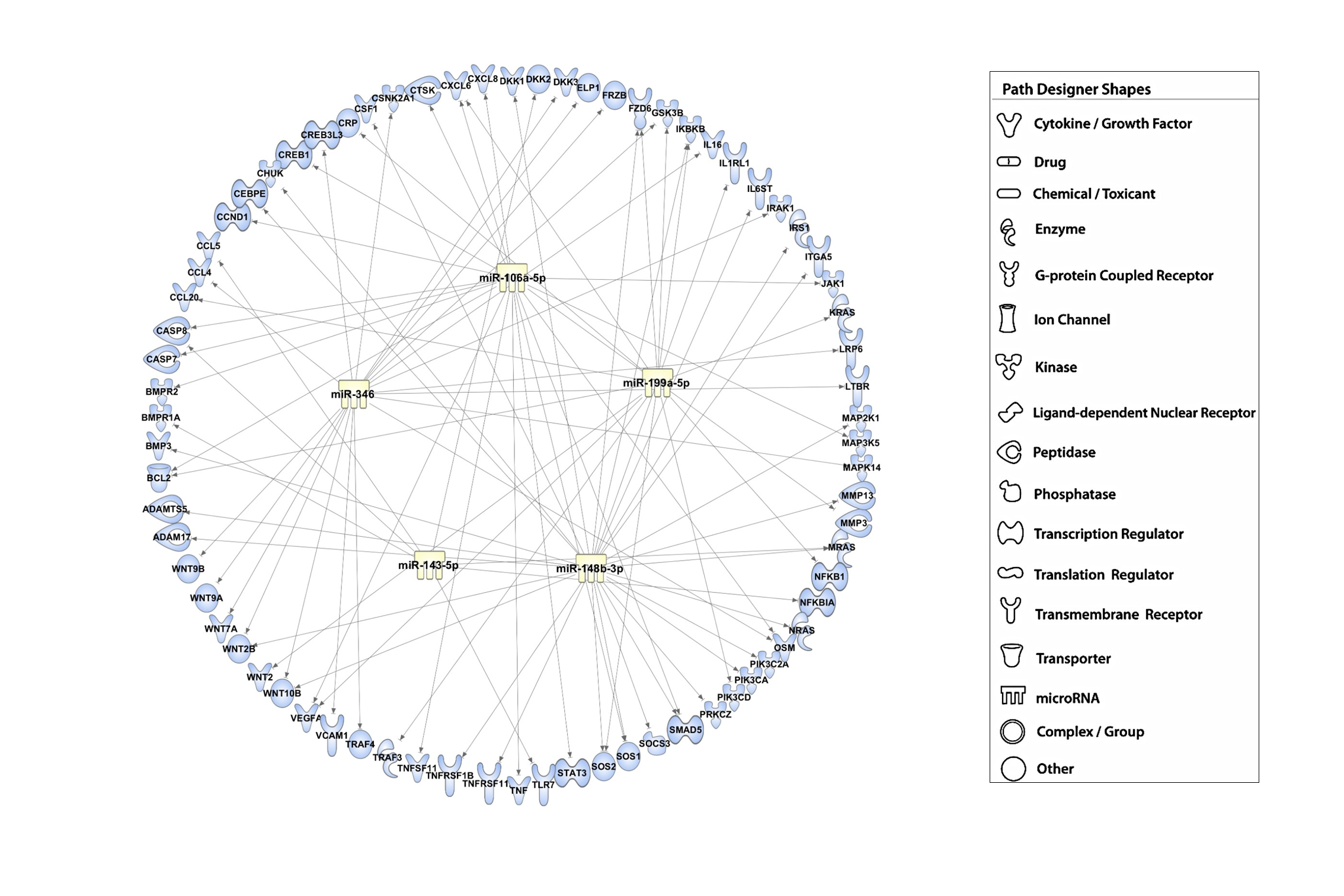


**Supplemental Figure 2**.  **Interaction network of microRNAs and their potential mRNA targets involved in key clinical features of Rheumatoid Arthritis**. The microRNA Target Filter of QIAGEN’s Ingenuity Pathway Analysis software generated a network including the five selected microRNAs and their potential mRNA targets, filtered by Rheumatoid Arthritis pathways. Targets experimentally observed and predicted with high confidence are shown and related to their specific microRNA regulators

**Supplemental Figure 3.** I**nteractions among miRNAs and their pro-inflammatory target genes.** A) Interactions between hsa-miR-106a-5p and its pro-inflammatory targets. B) Interactions between hsa-miR-148b-3p and its pro-inflammatory targets. C) Interactions between hsa-miR-143-5p and its pro-inflammatory targets. D) Interactions between hsa-miR-199a-5p and its pro-inflammatory targets. E) Interactions between hsa-miR-346 and its pro-inflammatory targets.

**
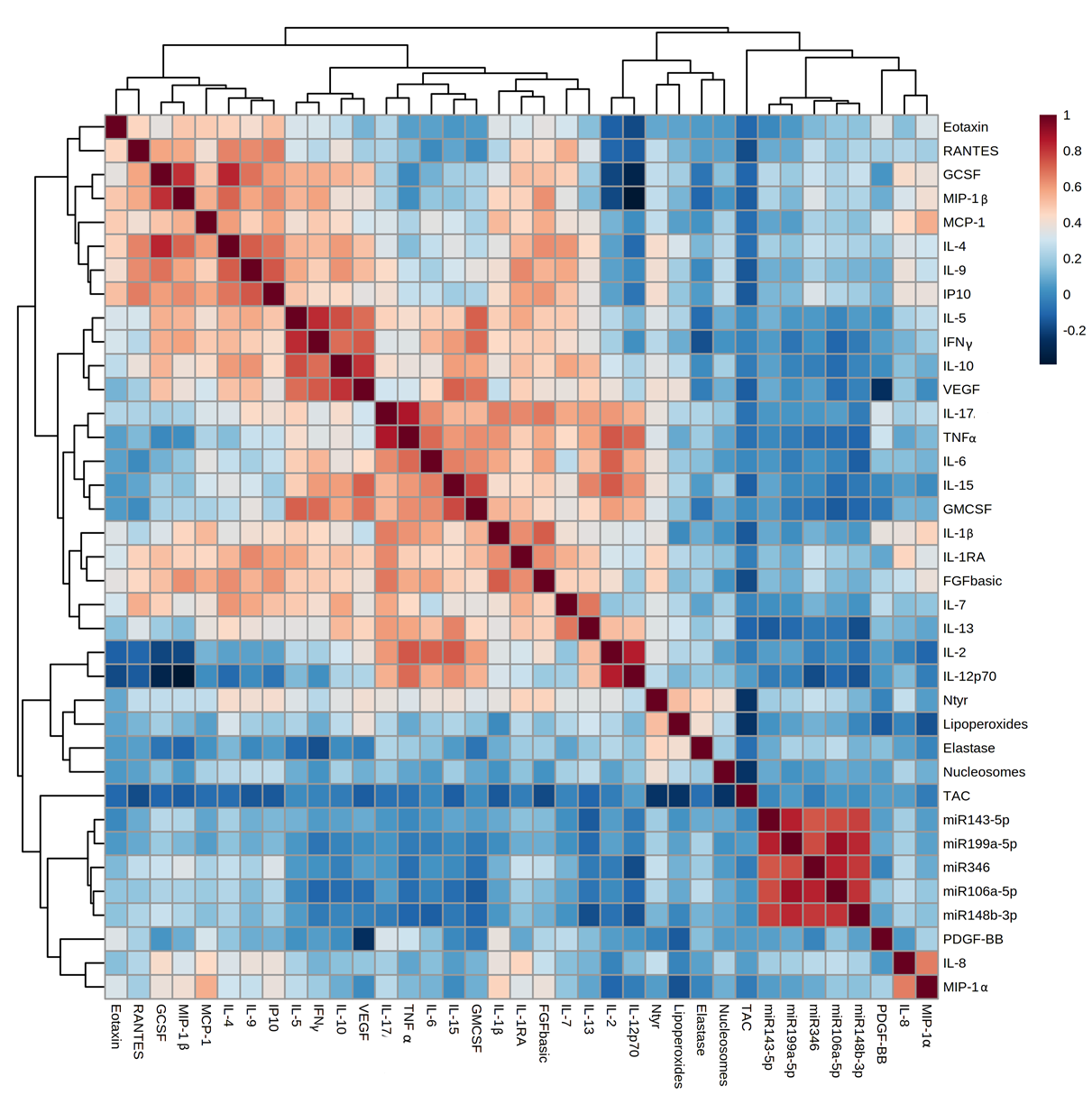
**

**Supplemental Figure 4. Coordinated alteration of molecular parameters analysed in the plasma of Rheumatoid Arthritis patients.** Heat map of correlation among the biomolecules analysed in the plasma of Rheumatoid Arthritis patients. Red color indicates positive correlations while blue color shows negative correlations.

**Supplemental Figure 5. Similar pattern changes in TNFi types.** A) Changes after 6 months of TNFi therapy in clinical parameters in monoclonal antibodies versus receptor soluble. A) Changes after 6 months of TNFi therapy in the molecular parameters analyzed in monoclonal antibodies versus receptor soluble.

**Supplemental Figure 6. Similar prediction patterns in monoclonal antibodies versus soluble receptor.** ROC curve of machine learning clinical, molecular, and mixed models predictors of clinical response in RA patients treated with monoclonal antibodies (left panels) or soluble receptor (right panels). AUC, area under the curve.

**Supplementary Tables**

**Supplementary Table 1.** Clinical and molecular profile of Rheumatoid Arthritis patients recruited as an independent validation cohort.

**Supplementary Table 2.** miRNome profile of Rheumatoid Arthritis patients and healthy donors.

| **Serum from RA patients vs HD** | | | | | | | | | |
| --- | --- | --- | --- | --- | --- | --- | --- | --- | --- |
| **microRNAs** | **Fold change** | **microRNAs** | **Fold change** | **microRNAs** | **Fold change** | **microRNAs** | **Fold change** | **microRNAs** | **Fold change** |
| miR-451a | -37,49 | miR-106b-3p | -7,69 | miR-6726-5p | 2,02 | miR-548j-3p | 2,42 | miR-4728-3p | 3,8 |
| miR-20a-5p | -25,67 | miR-19a-3p | -7,67 | miR-6744-3p | 2,02 | miR-6789-5p | 2,43 | miR-548ay-5p | 3,8 |
| miR-144-3p | -24,71 | miR-140-5p | -7,53 | miR-6789-3p | 2,02 | miR-2116-3p | 2,48 | miR-1269b | 3,84 |
| miR-16-5p | -24,62 | miR-30c-5p | -7,46 | miR-1247-3p | 2,05 | miR-6862-3p | 2,48 | miR-566 | 3,84 |
| miR-15b-5p | -23,78 | miR-101-3p | -7,21 | miR-561-3p | 2,05 | miR-4539 | 2,49 | miR-548b-5p | 3,88 |
| miR-199a-3p | -19,69 | miR-150-5p | -6,91 | miR-5587-3p | 2,07 | miR-937-5p | 2,49 | miR-574-5p | 4,01 |
| miR-106b-5p | -16,5 | miR-145-5p | -6,84 | miR-5694 | 2,07 | miR-1306-5p | 2,51 | miR-548e-5p | 4,04 |
| miR-15a-5p | -15 | miR-29c-3p | -6,79 | miR-640 | 2,09 | miR-6870-3p | 2,51 | miR-1273g-5p | 4,12 |
| miR-93-5p | -14,88 | miR-222-3p | -6,76 | miR-6511a-3p | 2,09 | miR-4447 | 2,52 | miR-574-3p | 4,12 |
| miR-19b-3p | -14,57 | miR-130b-3p | -6,26 | miR-6892-3p | 2,09 | miR-4535 | 2,53 | miR-1914-5p | 4,24 |
| miR-17-5p | -14,26 | miR-126-3p | -6,05 | miR-4498 | 2,1 | miR-1202 | 2,55 | miR-1322 | 4,34 |
| miR-25-3p | -13,63 | miR-652-3p | -6,05 | miR-449a | 2,1 | miR-6722-3p | 2,57 | miR-4421 | 4,35 |
| miR-223-3p | -13,61 | miR-181a-5p | -5,71 | miR-6852-3p | 2,1 | miR-8071 | 2,57 | miR-4496 | 4,49 |
| miR-26b-5p | -13,33 | let-7b-5p | -5,67 | miR-6884-3p | 2,11 | miR-1231 | 2,59 | miR-6500-3p | 4,53 |
| miR-361-5p | -12,81 | let-7e-5p | -5,62 | miR-2116-5p | 2,12 | miR-6765-5p | 2,6 | miR-1304-3p | 4,63 |
| miR-6798-5p | -12,39 | miR-376a-3p | -5,46 | miR-381-3p | 2,13 | miR-1249 | 2,61 | miR-1254 | 4,78 |
| miR-27a-3p | -12,15 | miR-29a-3p | -5,05 | miR-3912-5p | 2,13 | miR-658 | 2,63 | miR-1303 | 4,78 |
| miR-130a-3p | -12,04 | miR-127-3p | -5,02 | miR-187-5p | 2,15 | miR-3670 | 2,64 | miR-1273a | 4,83 |
| miR-142-5p | -11,67 | miR-24-3p | -4,91 | miR-4695-5p | 2,15 | miR-6825-3p | 2,68 | miR-1273e | 4,96 |
| miR-26a-5p | -11,48 | miR-27a-5p | -4,67 | miR-4769-3p | 2,15 | miR-6802-5p | 2,74 | miR-1273c | 5,11 |
| let-7d-5p | -11,25 | let-7c-5p | -4,54 | miR-764 | 2,15 | miR-8078 | 2,74 | miR-548w | 5,12 |
| miR-6752-5p | -11,25 | miR-331-3p | -4,37 | miR-6723-5p | 2,16 | miR-4512 | 2,76 | miR-1273h-5p | 5,19 |
| miR-23a-3p | -10,75 | miR-193a-5p | -4,22 | miR-4722-3p | 2,17 | miR-548ax | 2,78 | miR-1273d | 5,38 |
| miR-106a-5p | -10,51 | miR-378d | -3,84 | miR-6511b-3p | 2,17 | miR-3127-3p | 2,81 | miR-649 | 5,46 |
| miR-191-5p | -10,49 | miR-28-5p | -3,39 | miR-1539 | 2,18 | miR-4461 | 2,82 | miR-5585-3p | 5,56 |
| let-7a-5p | -10,28 | miR-484 | -3,36 | miR-204-3p | 2,18 | miR-5739 | 2,82 | miR-1299 | 5,83 |
| let-7i-5p | -10,21 | miR-425-3p | -3,2 | miR-877-3p | 2,18 | miR-6809-5p | 2,82 | miR-3674 | 6,05 |
| miR-199a-5p | -10,13 | miR-192-5p | -3,15 | miR-4725-5p | 2,19 | miR-34b-3p | 2,9 | miR-3135a | 6,19 |
| miR-185-5p | -9,76 | miR-125a-5p | -2,77 | miR-1207-5p | 2,22 | miR-6882-3p | 2,9 | miR-1285-5p | 6,24 |
| miR-148a-3p | -9,74 | miR-324-5p | -2,69 | miR-187-3p | 2,22 | miR-6787-5p | 2,92 | miR-1255b-2-3p | 6,35 |
| miR-103a-3p | -9,73 | miR-6782-5p | -2,69 | miR-6819-3p | 2,22 | miR-6804-3p | 2,93 | miR-548d-5p | 7,06 |
| miR-342-3p | -9,59 | miR-128-3p | -2,65 | miR-6877-3p | 2,22 | miR-1244 | 2,98 |  |  |
| miR-30b-5p | -9,41 | miR-2110 | -2,48 | miR-3912-3p | 2,23 | miR-4763-3p | 2,98 |  |  |
| miR-425-5p | -9,31 | miR-410-3p | -2,4 | miR-4723-3p | 2,23 | miR-1915-3p | 3,02 |  |  |
| miR-221-3p | -9,3 | miR-22-5p | -2,24 | miR-766-3p | 2,23 | miR-3937 | 3,02 |  |  |
| miR-107 | -9,18 | miR-103a-2-5p | -2,04 | miR-146b-3p | 2,25 | miR-3663-5p | 3,06 |  |  |
| miR-18a-5p | -9,18 | miR-378e | -2,04 | miR-4426 | 2,25 | miR-1227-3p | 3,07 |  |  |
| miR-22-3p | -9,09 | miR-7-1-3p | -2,04 | miR-561-5p | 2,25 | miR-1228-3p | 3,23 |  |  |
| miR-148b-3p | -9,02 | miR-212-3p | 2 | miR-210-5p | 2,26 | miR-1225-3p | 3,29 |  |  |
| miR-27b-3p | -9,02 | miR-346 | 2 | miR-4795-3p | 2,27 | miR-4502 | 3,3 |  |  |
| miR-143-3p | -8,89 | miR-3907 | 2,01 | miR-1229-3p | 2,29 | miR-548h-5p | 3,35 |  |  |
| miR-92a-3p | -8,65 | miR-5006-5p | 2,01 | miR-6775-3p | 2,3 | miR-5684 | 3,37 |  |  |
| let-7f-5p | -8,48 | miR-876-3p | 2,01 | miR-3184-3p | 2,33 | miR-297 | 3,42 |  |  |
| miR-301a-3p | -8,17 | miR-143-5p | 2,01 | miR-6792-3p | 2,35 | miR-3934-5p | 3,52 |  |  |
| miR-18b-5p | -8,15 | miR-1237-3p | 2,02 | miR-1234-3p | 2,37 | miR-1287-5p | 3,53 |  |  |
| miR-363-3p | -8,1 | miR-1910-5p | 2,02 | miR-1913 | 2,39 | miR-5698 | 3,55 |  |  |
| miR-23b-3p | -7,79 | miR-449b-3p | 2,02 | miR-4713-3p | 2,39 | miR-616-3p | 3,56 |  |  |
| miR-486-5p | -7,72 | miR-541-3p | 2,02 | miR-548ak | 2,4 | miR-548az-5p | 3,67 |  |  |
